# High-Sensitivity Cardiac Troponin Concentrations at Presentation in Patients With ST-Segment Elevation Myocardial Infarction

**DOI:** 10.1001/jamacardio.2020.2867

**Published:** 2020-08-12

**Authors:** Ryan Wereski, Andrew R. Chapman, Ken K. Lee, Stephen W. Smith, David J. Lowe, Alasdair Gray, Nicholas L. Mills

**Affiliations:** 1 BHF Centre for Cardiovascular Science, University of Edinburgh, Edinburgh, Scotland; 2 University of Minnesota, Emergency Medicine, Minneapolis; 3 University of Glasgow, School of Medicine, Glasgow, Scotland; 4 Royal Infirmary of Edinburgh, Emergency Medicine Research Group, Edinburgh, Scotland

## Abstract

This secondary analysis of a randomized clinical trial evaluates the testing of high-sensitivity cardiac troponin in clinical practice.

The introduction of high-sensitivity cardiac troponin testing into clinical practice has transformed the assessment of patients with suspected acute coronary syndrome in the emergency department.^1^ Most patients can be discharged using accelerated diagnostic pathways that do not require hospital admission for peak cardiac troponin testing.^2^ These pathways are not recommended for patients with ST-segment elevation on the electrocardiogram,^3,4^ but given that interpretation is dependent on experience, there is a risk patients could be inappropriately assessed.

## Methods

Between June 2013 and March 2016, consecutive patients with suspected acute coronary syndrome were recruited across 10 hospitals in the High-Sensitivity Troponin in the Evaluation of Patients With Acute Coronary Syndrome (High-STEACS) cluster randomized clinical trial.^5^ High-sensitivity cardiac troponin I was measured using the Abbott ARCHITECT STAT assay (Abbott Laboratories), which has a limit of detection of 1.2 ng/L, and a 99th percentile upper reference limit of 34 ng/L in men and 16 ng/L in women. The index diagnosis was independently adjudicated by 2 physicians on review of all clinical information, according to the Universal Definition of Myocardial Infarction.^6^ Patients with type 1 ST-segment elevation myocardial infarction (STEMI) were stratified according to cardiac troponin concentration at presentation using a validated risk-stratification threshold (5 ng/L),^1^ the sex-specific 99th percentile, and the European Society of Cardiology (ESC) 0 of 1 hour pathway rule-in threshold (52 ng/L).^4^ Posterior STEMI was defined as those with STEMI and an acute occlusion of the circumflex, obtuse marginal, or posterior left ventricular artery on angiography. Time from symptom onset was recorded prospectively by attending clinicians. The trial was approved by the National Health Service Scotland A Research Ethics Committee, the Public Benefit and Privacy Panel for Health and Social Care, and by each local National Health Service Health Board. Because randomization was at the hospital level, individual patient consent was not sought.^5^ Comparisons between groups were performed using the χ^2^ test for categorical variables and an unpaired *t* test or the Kruskal-Wallis test for continuous variables. Statistical analysis was performed using R, version 3.6.1 (R Foundation).

## Results

The trial enrolled 48 282 consecutive patients, of whom 925 had an adjudicated diagnosis of STEMI (67.8% men [n = 627 of 925]; mean [SD] age, 65 [14] years). At presentation, the median troponin concentration was 196 ng/L (interquartile range [IQR], 46-21 611 ng/L), with 2.2% (n = 20 of 925) and 14.4% of patients (n = 133 of 925) having concentrations less than 5 ng/L and the 99th percentile, respectively (Figure). Just 73.2% of patients (n = 677 of 925) had troponin concentrations greater than the rule-in threshold of 52 ng/L. Patients presenting within 2 hours of symptom onset (23.4%; 216 of 809) had lower troponin concentrations (96 ng/L; IQR,26-494 ng/L vs 294 ng/L; IQR, 59-3042 ng/L; *P* < .001) and were more likely to have concentrations at less than the 99th percentile (26.4% [n = 57 of 216] vs 14.1% [n = 95 of 674]; *P* < .001), compared with those presenting later. Posterior STEMI was more common in patients presenting with troponin at less than the 99th percentile (18.1% [n = 26 of 144] vs 9.8% [n = 61 of 618]; *P* = .008).

**Figure.**
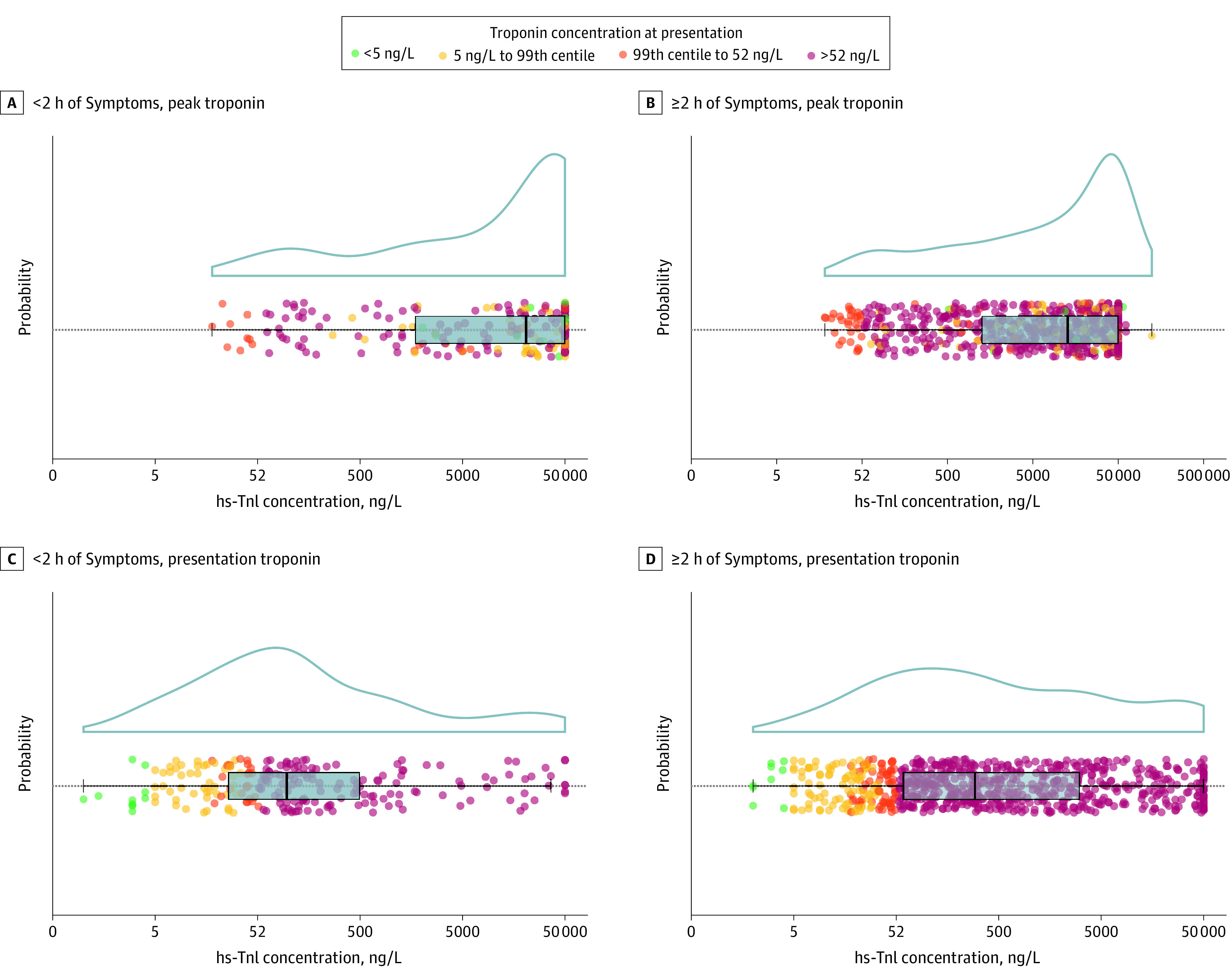
High-Sensitivity Cardiac Troponin I (hs-TnI) Concentrations in Consecutive Patients With ST-Segment Elevation Myocardial Infarction Stratified by Troponin Concentration at Presentation Patients were stratified by time of onset of symptoms (<2 hours and ≥2 hours) and according to cardiac troponin concentration at presentation. Individual patient concentrations (less than the rule-out threshold of <5 ng/L [green; to convert to micrograms per liter, multiply by 1], 5 ng/L to the 99th percentile diagnostic threshold [yellow], 99th percentile to 52 ng/L rule-in threshold for the ESC 0/1 hours pathway [orange], and >52 ng/L [red]) are shown with box and whisker distribution and probability density plots. Peak concentration is the highest troponin concentration obtained on serial sampling.

## Discussion

Despite significant advances in the sensitivity of cardiac troponin testing, more than 1 in 4 patients with STEMI have troponin concentrations at less than the ESC-recommended rule-in threshold at presentation. Patients presenting within 2 hours were more likely to have a troponin concentration at less than the 99th percentile; however, even in those who presented later, 1 in 6 had troponin concentrations at less than the diagnostic threshold. During myocardial infarction, abrupt coronary occlusion may prevent the release of troponin into the circulation until reperfusion has occurred. Our observations are an important reminder of the limited role of troponin testing in the early assessment of patients with ST-segment elevation. Where clinical suspicion is high, troponin concentrations within the reference range should not delay the initiation of therapeutic agents or urgent coronary angiography. This is particularly relevant in patients with electrocardiographic changes suspicious of posterior myocardial infarction, but our findings are relevant to a wider group of patients with conduction abnormalities, such as bundle branch block or ventricular pacing, where interpretation of the electrocardiogram is challenging.
